# The Importance of Dosimetry Standardization in Radiobiology

**DOI:** 10.6028/jres.118.021

**Published:** 2013-12-30

**Authors:** Marc Desrosiers, Larry DeWerd, James Deye, Patricia Lindsay, Mark K Murphy, Michael Mitch, Francesca Macchiarini, Strahinja Stojadinovic, Helen Stone

**Affiliations:** 1 National Institute of Standards and Technology, Gaithersburg, Maryland 20899; 2 University of Wisconsin School of Medicine and Public Health, Madison, Wisconsin; 3 National Cancer Institute, National Institute of Health, Bethesda, Maryland; 4 Princess Margaret Hospital, University of Toronto, Toronto, Ontario, Canada; 5 Battelle–Pacific Northwest National Laboratory, Richland, Washington; 6 National Institute of Allergy and Infectious Diseases, National Institutes of Health, Bethesda, Maryland; 7 University of Texas Southwestern Medical Center Dallas, Texas

**Keywords:** dosimetry, dosimetry protocols, dosimetry standards, radiobiology, radiobiology protocols, radiobiology standards

## Abstract

Radiation dose is central to much of radiobiological research. Precision and accuracy of dose measurements and reporting of the measurement details should be sufficient to allow the work to be interpreted and repeated and to allow valid comparisons to be made, both in the same laboratory and by other laboratories. Despite this, a careful reading of published manuscripts suggests that measurement and reporting of radiation dosimetry and setup for radiobiology research is frequently inadequate, thus undermining the reliability and reproducibility of the findings. To address these problems and propose a course of action, the National Cancer Institute (NCI), the National Institute of Allergy and Infectious Diseases (NIAID), and the National Institute of Standards and Technology (NIST) brought together representatives of the radiobiology and radiation physics communities in a workshop in September, 2011. The workshop participants arrived at a number of specific recommendations as enumerated in this paper and they expressed the desirability of creating dosimetry standard operating procedures (SOPs) for cell culture and for small and large animal experiments. It was also felt that these SOPs would be most useful if they are made widely available through mechanism(s) such as the web, where they can provide guidance to both radiobiologists and radiation physicists, be cited in publications, and be updated as the field and needs evolve. Other broad areas covered were the need for continuing education through tutorials at national conferences, and for journals to establish standards for reporting dosimetry. This workshop did not address issues of dosimetry for studies involving radiation focused at the sub-cellular level, internally-administered radionuclides, biodosimetry based on biological markers of radiation exposure, or dose reconstruction for epidemiological studies.

## 1. Introduction

The goal of much of radiobiology research is to establish the relationship between radiation dose and the magnitude of the effect of that dose. While standards for acquiring and reporting dose distributions in clinical radiotherapy have risen rapidly over the past 20 years, dosimetry for research in radiobiology in published papers is frequently inadequately described, which suggests that the dosimetry itself may have been inadequate. This is particularly a concern when there is a steep dose-response relationship, when data among different laboratories is to be compared, and when researchers try to repeat experiments. A workshop was therefore convened in September, 2011, by the National Cancer Institute (NCI), the National Institute of Allergy and Infectious Diseases (NIAID), and the National Institute of Standards and Technology (NIST) with two primary goals. The first was to assess the current status and highlight the importance of dosimetry standardization in radiobiology research, whether it involves *in vitro* (cell culture) or *in vivo* approaches in small or large animal models. The second longer-term goal was to address the needs for developing a formal system for coordinating standardization efforts as well as establishing a continuing education series through tutorials at national conferences, publications, and web-based resources.

To determine whether a more focused and organized effort in dosimetry standardization could significantly impact the field of radiobiology, it is important to consider what the current needs are in the field with regard to dosimetry precision and accuracy and, second, whether most researchers are meeting these needs. One can then assess whether most researchers are able, and have the resources, to accurately quantify the dose and the associated uncertainty of their dose measurements and calculations.

In addition to the need for standardization of dosimetry within and between laboratories, consideration was given to the dosimetry detail required within radiobiology publications. Publications that lack critical detail on dosimetry make it difficult or impossible for other researchers to repeat or to make valid comparisons with these studies.

Finally, the workshop considered the coverage of radiation dosimetry for radiobiology experiments within publications by reference organizations such as the International Commission on Radiation Units and Measurements (ICRU), International Atomic Energy Association (IAEA), and the American Association of Physicists in Medicine (AAPM), and the acknowledgment of such standards within the literature.

These issues were examined during the workshop through presentations and discussions among experts in radiobiology, radiation physics and dosimetry, editors of relevant journals and metrologists. (See: http://www.nist.gov/pml/div682/grp02/dosimetry-standardization-for-radiobiology.cfm).

This manuscript summarizes the broad points of discussion during the workshop as well as the recommendations that were addressed to the radiobiology community. It is expected that specific details concerning the three broad areas of radiobiology experiments: *in vitro*, small and large *in vivo* irradiations will be dealt with in subsequent manuscripts.

## 2. Accuracy of Dose Versus Precision (or Reproducibility) of Dose

**Fig. 1:**
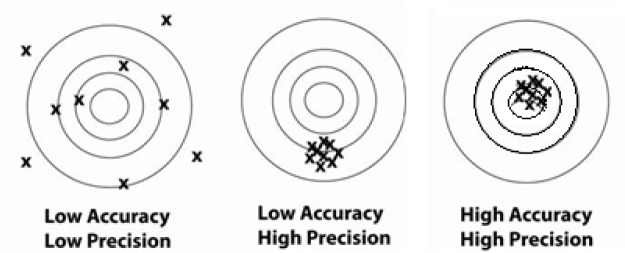
*Accuracy* indicates proximity of measurement results to the true (target) value, while *precision* indicates the repeatability, reproducibility or spread of the measurement.

When planning any radiobiology study, one needs to decide the level of precision and accuracy required for the radiation dose. Figure 1 illustrates that *accuracy* indicates agreement (proximity) of measurement results to the true value (target center), and *precision* refers to the repeatability or reproducibility of the measurement (distribution of measurements). Many researchers emphasize reproducibility (precision) of dose within their own lab results without giving due consideration to accuracy of the reported dose value (comparison to the primary standard of dose). They try to minimize the size of the error bars by concentrating on the local result while employing a minimal amount of data. However, what is typically called error bars is actually the standard deviation (i.e. the deviation from the mean) which only approaches the true error if a very large number of measurements are made and if systematic errors have been analyzed and adequately addressed. Such precise local measurements may suffice to test the sensitivity of a biological endpoint to radiation dose. However, if dose is inaccurate, then the conclusions may be invalid or not reproducible in other labs, and treating a biological sample with an inaccurate dose may result in a response that is not correctly linked to the pertinent biological radiation interaction. All the experiments must be performed using the same (accurate) measurement of dose.

Figure 1 portrays that, while precision may be reasonable (as in the middle target frame), the deviation from the true value (target center) may be larger than the spread of the measurements. Hence the value of dose that would be compared to that obtained by other researchers could be off by a multiple of the spread (precision) of the measurements.

## 3. Precision and Accuracy Requirements in Radiobiology Research

In order to determine whether increasing standardization of dosimetry in radiobiology could significantly impact the field, consideration was first given to the current needs in the field with regard to dosimetry precision and accuracy for many of the biological endpoints for *in vitro* and *in vivo* irradiations, such as: survival curves, apoptosis, gene or protein expression, mutations, cell transformation, functional changes, etc.

**Fig. 2:**
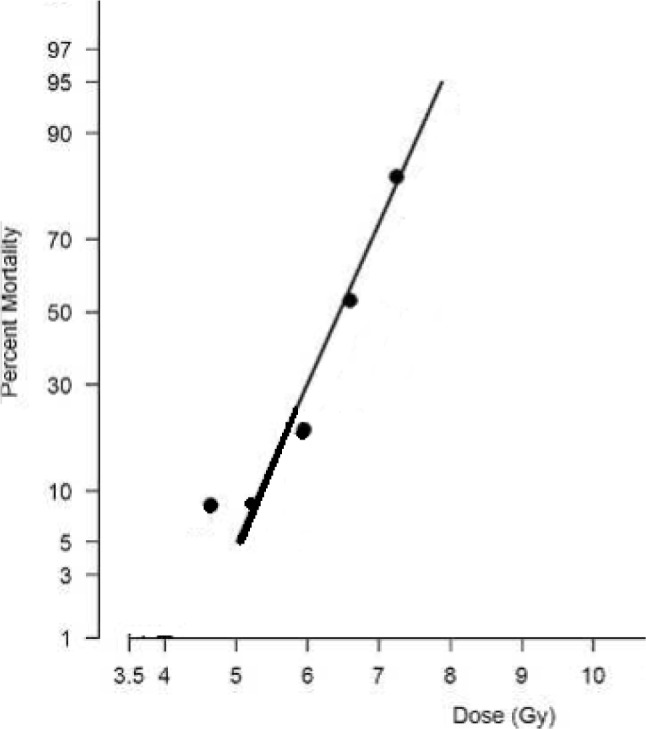
An example of a typical dose-response relationship for total-body-irradiated (TBI) non-human primates. The 60-day mortality dose-response relationship is presented as probit percent mortality versus TBI dose (Gy) on a linear scale.

**Fig. 3:**
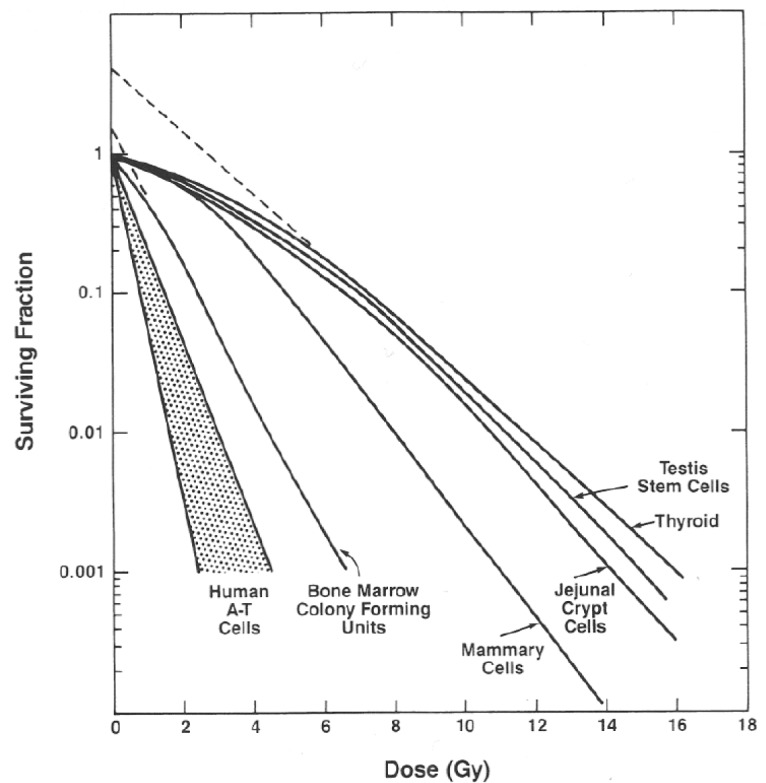
High dependence of cell survival on dose for different cell types. [Provided by Elizabeth Travis, personal communication.]

Biological dose-response variability can be very large even within a single species. This wide range in dose response is influenced by genetic sensitivity as well as environmental factors that influence response to radiation. For molecular responses as well, the data suggest that there is tremendous dose response variability within tissues, species, strains, and cell types. The result is that many radiobiology studies do not require precision of absorbed dose across the study group of more than perhaps 10 %. However, some biological endpoints are very sensitive to the change in the radiation dose and, even when controlling other experimental variables, require much better than 10 % precision in dose to resolve both dose-related and non-dose-related influences. Examples are induction of myelopathy following spinal cord irradiation, GI injury, bone marrow, lung responses and lethality (Fig. 2) and cell survival curves (Fig. 3). The steepness of these lethality curves demonstrates the need for precision within study groups, and the ability to intercompare the different groups requires accuracy in the stated radiation doses that is at least equal to the precision of the measurements. Both Figs. 2 and 3 illustrate that a precise and accurate measurement of dose is critical if the rate of change of the biological effect is to be observed.

In the irradiation of humans, Report 24 (1976) by the ICRU [1] stated that a change of 7 % to 10 % in dose to target volume results in a clinically significant change in the tumor control probability, which led to a consensus in the radiation oncology community that absorbed dose delivered to the tumor volume should be within 5 % of the prescribed dose.

## 4. Factors that Govern Radiation Dose and its Distribution

Since it is recognized that many biologists working with radiation have not had in-depth training in the principles of radiation physics, the following is meant to be a very brief outline of important physics principles that are to be considered in the design of radiation biology experiments.

**Fig. 4:**
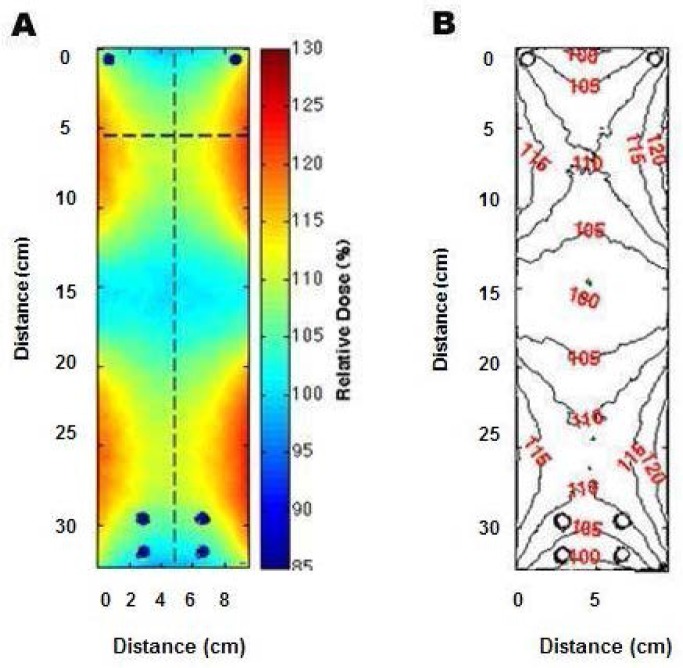
Dose variation in a Cs-137 irradiator shown with a) dose color wash and b) isodose mapping of the +25 % and −15 % variation in dose throughout the irradiation volume [2].

Radiation sources must be characterized in terms of their physical size, energy and dose rate along with the factors that can affect these characteristics:

Depth dose, buildup and falloff of dose with depth in medium or tissue:
  - depends on type and energy of radiation beam
  - depends on composition of tissue: e.g., bone vs. soft tissue
Distance from source:
  - affects dose rate if different from that used for dosimetry
  - affects ratio of primary radiation to scattered radiation
  - affects dose falloff through sample
Radiation field size:
  - affects the flatness of the radiation field since the edges of a large field are at a greater distance from the radiation source than the center of the field, which results in a lower dose rate at the edges than at the field center
  - causes dose rate changes with the change in field size due to variations in the amount of scatter
  - contributes to a lower effective energy at the field edges due to a higher proportion of scatter radiation
Filters that are in the radiation field in order to “harden” the beam:
  - have a major impact on the effective energy of the beam
  - significantly reduce the dose rate
Use of any materials or sample containers in the beam, even those that are under or beyond the irradiated sample or animal must be accounted for in dosimetry:
  - for their beam attenuation
  - for their side and back-scatter onto the sample
Even self-contained fixed-source irradiators exhibit significant variations in dose within the sample chamber (see Fig. 4).

Further detailed discussions of each of these factors can be found in most standard textbooks on radiation physics, including those listed in Refs. [3–7].

## 5. The Role of Primary and Secondary Standards Laboratories

Traceability of radiation dose is dependent on standards that are the same throughout the world. This requires primary laboratories such as the NIST in the United States to maintain reference standards for measureable quantities and set procedures for transferring these standards to the communities that need to measure the subject quantities. Thus, NIST maintains the U.S. standard for radiation dose and transfers this standard through sanctioned secondary calibration laboratories: Accredited Dosimetry Calibration Laboratories (ADCLs). A facility that desires to perform irradiations must ensure that its dosimetry equipment is properly characterized and calibrated by a NIST traceable lab, that the radiation source(s) it will use are likewise calibrated with NIST traceability and that the procedures that it will use are well documented and standardized. Note that the term traceability refers to the “property of a measurement result whereby the result can be related to a reference through a documented unbroken chain of calibrations, each contributing to the measurement uncertainty” as stated in the International Vocabulary of Metrology — Basic and General concepts and Associated Terms (VIM), definition 2.41.

NIST traceability therefore allows an uncertainty to be assigned to the dose under calibration conditions, which forms the basis to evaluate the uncertainty of the delivered dose for a given experiment.

## 6. Current Status of Radiobiology Dosimetry

The workshop participants examined current realities of radiobiology dosimetry from two major perspectives, as practiced in the laboratory and as reported in the literature. There was concern that researchers can’t report what they haven’t considered. The field suffers even if all factors are properly taken into account but not reported in the literature, leaving readers uncertain of the reliability of the data.

**Fig. 5:**
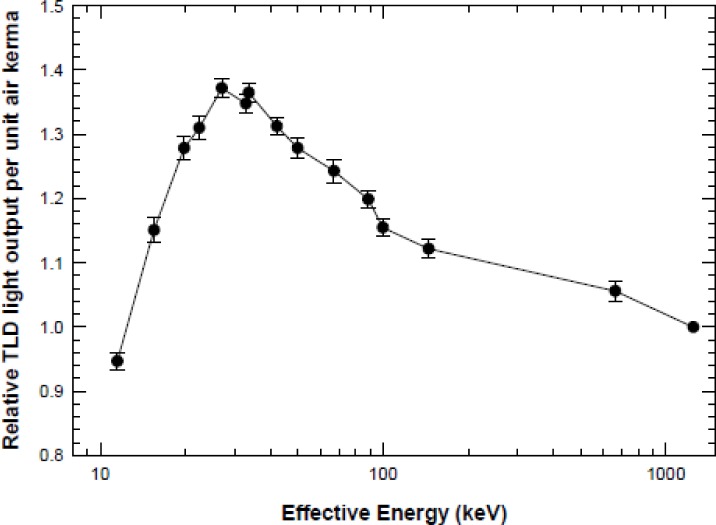
Energy dependence of thermoluminescent dosimeter (TLD) response [9].

**Fig. 6:**
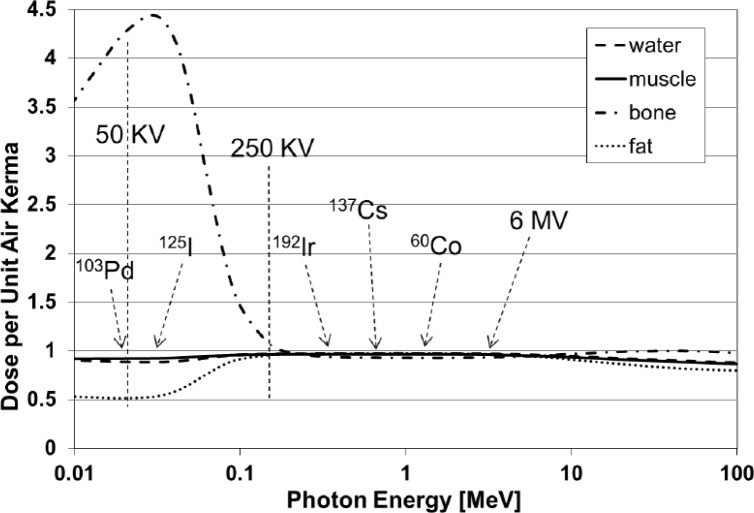
Variation of dose in water, muscle, bone and fat per unit of air kerma (proportional to exposure) at various energies. [Provided by William Hanson, personal communication.]

- In the lab:
  A number of examples were presented of large dose variations that can result when users of radiation devices were not fully aware of their characteristics, especially those resulting from source-sample distances, energy characterization and variation, field size, and differences between calibration and sample irradiation conditions.
  - An early report from the Armed Forces Radiobiology Research Institute (AFRRI) (AFRRI TR89-1) observed that “the x-ray energy spectrum produced at a peak voltage of 50 kV and with added Al filters readily undergoes attenuation by the plastic tissue-culture Petri-dish covers or the culture media. For example, using a beam hardened with 0.18 mm of Al, the attenuation due to the medium can be as high as 60 % and the plastic cover will reduce the beam an additional 15 %.”
  - Manufacturer-supplied calibrations for a number of commercially-available irradiators have been found to differ by +5 % to −13 % from their true values with variations in dose rate over irradiation volumes from 70 % to 180 % of the stated value. (Ref. [8] and also Fig. 4.)
  - An inter-laboratory comparison of dosimetry for animal irradiation conducted by an NIAID-funded multi-site project found that some measured results differed from the expected values over a range of −2 % to −26 % as a result of the energy dependence of the dosimeter measurement system being used. Figure 5 depicts the measured energy dependence of commonly used thermoluminescent dosimeters (TLD) as published by Nunn et al. [9].
  - Chow et al. [10], using Monte Carlo calculations for 100 kV and 225 kV x-ray beams, demonstrated that mouse bone had over 400 % greater dose than that in water or soft tissue at these radiation energies because of the greater absorption of lower-energy radiation by bone, a property exploited for diagnostic x-rays (see also Fig. 6). Thus it is important to account for the actual physical interactions of the radiation with the biological sample in a realistic way if the true doses to biological tissues are to be accurately reported.
  Despite these and numerous other examples, many radiobiology experimenters do not routinely employ a radiation physicist in the design or implementation of their experiments, and they rely on historical or vendor-supplied data for the determination of the sample doses. Few students or researchers using ionizing radiation in biological research have training in basic radiation physics. This leads to the difficult situation that when “one does not know what one does not know,” dosimetry design and documentation go unaddressed.
  Even when radiobiologists look for radiation physics consultation, it has become more difficult to achieve because the workloads of clinical medical physicists have increased significantly and the equipment that is used for radiobiology is seldom used routinely in the clinic. Hence, the opportunities for such interactions are greatly diminished from what they were a number of years ago. In addition, most clinical physicists are no longer trained on the type of equipment that is used in radiobiology experiments and they may not have the requisite knowledge or appropriately calibrated measurement equipment (see Sec. 7) to characterize the irradiation experimental setup.
- In the literature:
  Many of the publications in the area of radiobiology studies contain minimal detail in irradiation geometry, radiation spectrum, measurement uncertainty, and dosimetry equipment and techniques. This can make it difficult for researchers to validly compare their radiobiology studies with other studies, design their study to reproduce another published study, and to determine how much of any discrepancy in biological response is due to absorbed dose delivered and how much is due to biological variation and analysis.
  Experiments that seek to determine the biological effects of radiation exposure must be grounded on accurate and precise dosimetry for that experiment, and publication of the results should include the following common parameters with their associated uncertainties:
  - Radiation field(s) to be used (e.g., radiation output, uniformity, energy)
  - Absorbed dose throughout the biological subject
  - Dose uniformity within the subject
  - Reproducibility of dose across a study group

Table 1 presents the approximate percentage of articles reporting specific dosimetric information in the journal Radiation Research between March, 2010 and March, 2011. These results indicate a significant lack of information in the areas of radiation geometry, dosimetry method, location of detector, dose reference location, uncertainty in the absorbed dose delivered, and the published standards or protocols used. Even this limited review raises serious concerns. For example, only 7 % of researchers cite written dosimetry standards/guides.

Though these examples are taken from a specific Journal, they are representative of the anecdotal experiences expressed by the experts at the workshop relating to a number of journals.

## 7. Meeting Federal Regulations or Conforming to National and International Standards

When results from radiobiology experiments are to be used in any regulatory setting, such as drug development, device approvals, or radiation safety standards, to name a few examples, it is imperative that the experiments be well designed and conditions documented so as to meet published requirements. The Food and Drug Administration (FDA) adheres to Good Laboratory Practices (GLP) as codified in 21 CFR 58 along with FDA Guidance Documents that can be found on their web site. An overarching GLP principle is “write it, do it, record it” which means if it isn’t documented it didn’t happen. For a study to be GLP compliant it must include adequate and permanent documentation of everything involved in an experimental test, including management plans and reviews of all aspects of the experiment, staff qualifications, validity of the study design, well-controlled environment, standard operating procedures, controlled collection and retention of study data, equipment calibration, validation and maintenance. These last few elements speak directly to the physical dosimetry of a radiobiology experiment.

The FDA has promulgated the Animal Rule (AR) along with a subsequent guidance documents [11] for the development of radiation-modifying drugs and/or medical procedures, and together these underscore the need for accurate dosimetry and well-defined radiation exposure protocols. As stated in Ref. [11] “Given the size of the error in the biological contribution, it is important that the physical errors are minimized. Furthermore, unless physical dose can be excluded as a variable in comparing studies, it is a waste to explore the source of the more complex biological variables that might contribute to differences in results between laboratories.”

Beyond regulatory concerns there is an increasing awareness of the high cost and inefficiencies resulting from inadequate standards for preclinical data in cancer research [12]. Whether studying radiation sensitizers or mitigators, it is clear that preclinical reproducibility is often lacking and that scientists and journal publishers must raise the bar for the design and reporting of such preclinical studies.

## 8. Equipment and Protocols for Radiobiology Dosimetry

Devices used to make measurements must be appropriate to the specifics of the irradiation procedures as described above. Examples of available dosimetry equipment along with their typical uses and response characteristics are listed in Table 2.

Uncertainty in measured dose can easily range from a few percent to well over an order of magnitude higher depending on the influence quantities related to conditions during and after irradiation and the knowledge of the person using the equipment. These quantities include: radiation energy, dose rate, temperature, relative humidity, dose fractionation, and factors related to irradiation geometry such as regions of dose heterogeneity, differences in tissue depth between dosimeter location and volume(s) of interest, as well as many others. Special attention should also be paid to the differences between the conditions used to calibrate the local irradiation device and the conditions of irradiation for a particular experiment, such as cells or small animals. For example, when calibration dosimetry is performed using clinical protocols, a 10 cm × 10 cm field size is often used with full scatter and homogeneous phantoms. However, during the irradiation of a small biological specimen (e.g., cell culture or mouse), the reduced scattering volume, differences in source-to-subject distance, output changes with field size, and other factors can lead to significant differences (> 20 %) in dose from that intended by the researcher. For radiobiology experiments involving irradiation energies in the kilo- and ortho-voltage ranges, the measurement device must be calibrated at the same energy as the experimental setup since both the dosimeters (Fig. 5) and biological specimens (Fig. 6) can have very large changes in response with beam energy.

In addition to real-time factors that can affect dosimeter response to radiation exposures, some non-direct-reading dosimeters can exhibit response changes after they have been irradiated. Post-irradiation influence quantities for such dosimeters include: time (growth/fading of dosimeter response after irradiation), storage temperature, conditioning treatment (*e.g*., some thermo-luminescent dosimeters require thermal annealing prior to measurement), relative humidity during storage, and exposure to light.

To assist in standardization of calibration methodologies, there have been a number of protocols published by national and international organizations. The following annotated sampling conveys the scope and relevance of some of the most noteworthy and widely used of such protocols. These may be very helpful in the design and execution of radiobiology experiments.

1. **ICRU 30 “Quantitative Concepts and Dosimetry in Radiobiology”** [13] is more comprehensive than most standards. Like TRS-398, it contains information on measuring accurate absorbed dose using ionization chambers but it also has a lot of information on survival curves, linear energy transfer (LET) and Lineal Energy, animal and cell culture exposure systems, scatter and charge particle equilibrium, along with recommended minimum dosimetric and irradiation geometry information required.
2. **AAPM TG 61 “40-300 kV X-ray Beam Dosimetry in Radiotherapy and Radiobiology”** [14] focuses on how to accurately measure absorbed dose of x-ray beams using ionization chambers in air or in water. Generally, the chambers are calibrated in terms of air kerma split into two major energy divisions (superficial and orthovoltage), centered around 100 keV.
3. **TG 51 and IAEA TRS-398 “Absorbed Dose Determination in External Beam Radiotherapy…”** [15–16] focuses on how to measure, traceably and accurately, absorbed dose in an external beam, in particular absorbed dose to water, whether for gamma ray, x-ray, Linac, electrons, or protons, whether using an ionization chamber in air or in water phantom. Generally, these two protocols are for megavoltage beams (i.e. energies greater or equal to that of Co-60) and use ionization chambers calibrated to absorbed dose to water. Various corrections that are needed to determine the absorbed dose to water, including differences in beam quality are provided in these protocols.

Other calibration protocols exist but one must use caution and ensure that they have not been superseded. A series of standard operating procedures will follow this manuscript to provide guidance on dosimetry standardization. Three manuscripts are planned on the standards and the dosimetry for: (a) cell cultures, (b) small animals, and (c) large animals.

## 9. Use of Dosimetry Comparison Programs

Dosimetry comparisons are relatively common in the medical and radiation protection dosimetry fields. Phantoms with imbedded dosimeters are sent to participating facilities with a detailed protocol for the irradiation(s). After exposure, the phantoms and dosimeters are returned to a facility that has NIST-traceable standards for analysis and comparison to expected results. These data can demonstrate the level of agreement among facilities as well as between each facility and the true expected dosimetry values. A similar program would be useful for the radiobiology community. For animal studies, the phantoms should approximate the shape and size of a mouse, rat, or other subject. Participants would be instructed to irradiate the phantom(s) according to a well-defined protocol and send the phantom(s) back to the central laboratory for analysis of the imbedded dosimeters. A comparison of cell culture irradiations could also be performed using actual petri dishes or well plates with water and water-tight dosimetry protection. Of course, each of these inter-comparison programs can be designed to look not only at the results of the detector readouts but also at other factors such as the participant’s interpretations of the irradiation protocols, the user’s understanding of the parameters that influence dose and dose distribution, along with their interpretation of data, and the NIST traceability and uncertainties associated with the measurements. For standardization of procedures, protocols should employ and reference publications such as those listed above as well as others from NIST and the AAPM. Workshop participants felt that dosimetry comparisons in the United States radiobiology research community in the past couple decades have been very limited at best, although there are several articles, going back to the early 1970s, reporting dosimetry comparisons in Europe and the United Kingdom performed under the European Radiation Dosimetry Group (EURADOS) and the European Late Effects Project Group (EULEP), but that there were none in the United States that resulted in a publication.

## 10. Multidisciplinary Needs

Given the highly specialized requirements for accurate radiation dosimetry, biologists and physicists must work together in the design, execution and interpretation of radiobiology experiments. This is especially important because:

1. Radiation equipment and methods are increasing in variety and complexity.
2. Radiation biologists rarely receive training in radiation dosimetry.
3. Radiation biologists usually use irradiation equipment dedicated to research that is not shared with and calibrated by their clinical colleagues.
4. Radiobiologists now rarely work with radiation physicists as part of their joint routine duties, and there are fewer radiation physicists who are trained in the unique characteristics of the equipment used and problems involved in performing dosimetry in support of radiation biology.

As with the collaboration between the biologist and statistician, which aids in determining the required sample size of the experiments, the biologist-physicist collaboration can aid in determining the accuracy and precision required by a given experimental design and the methods needed to achieve these. Consultation between the biologist and physicist can ensure an efficiently-designed experiment that uses appropriate equipment with reference to established protocols as well as appropriate interpretation of the observed results.

## 11. Recommendations

Though many issues were discussed, it became clear that certain themes recurred and that it is important to call attention to these. It is hoped that changes may result in the practices of the individual radiation biologists and their support personnel, as well as in the publication practices of the major journals in the field. These changes can be aided by additional work toward the setting and implementation of standards appropriate for radiation biology dosimetry and protocols.

In summary, the workshop participants put forward the following recommendations:

1. Biologists and physicists should collaborate on study design and execution.
2. Study design should indicate the accuracy and precision required to meet the expected experimental result.
3. A qualified radiation physicist should help to establish the methods needed to achieve the required accuracy and precision.
4. The physicist should help to establish an ongoing dosimetry constancy program with traceability to National or International standards.
5. Authors should include in their publications sufficient detail concerning the setup and dosimetry used for the study, including references to written standards and/or protocols used. This will require journal editors and reviewers to ensure compliance.
6. The radiobiology community should publish a list of the minimum dosimetry information to be included within publications (see examples in the Appendix).
7. The radiobiology community should determine where gaps exist in written standards and protocols and publish standards to fill those needs. The workshop participants recommended formation of 3 working groups tasked to develop protocols for routine radiobiology experiments: one each for cells, small laboratory animals, and large laboratory animals.
8. The radiobiology community should decide whether a formal dosimetry intercomparison program needs to be implemented for the radiobiology researchers and, if so, how will it be established and sustained.
9. One suggested mechanism for implementation of many of these recommendations would be to establish continuing education venues in both the radiobiology and physics communities to foster communication and arrive at agreed upon standards.

## Figures and Tables

**Table 1:** The approximate rate of occurrence of specific information within 15 issues covering March, 2010 through March, 2011, articles in the journal Radiation Research

Animal/Cell type 100 %	Dose (relative to water, tissue?) 94 %
Animal/Cell strain 100 %	Dose Rate (fractionated?) 81 %
Irradiator Manufacturer/Model 80 %	Location of Detector 20 %
Source (nuclide, HVL, filtering) 100 %	Dose Reference Location 7 %
Radiation Energy 78 %	Published Standards/Guides Used 7 %
Irradiation Geometry* 48 %	Uncertainty in Dose 4 %
Dosimetry Method 37 %	

* “TBI” or “PBI” were only given partial credit.

**Table 2:** Overview of dosimetry systems

Dosimeter Type	Applications	Advantages	Possible Disadvantages	Absorbed Dose Range	Uncertainty	Physical Resolution
**Air-filed Ionization Chambers**	•Radiation machine characterization (commissioning) •Absolute dose calibration (cGy/min, cGy/MU) •Quality Assurance (QA)	•Provides measurements traceable to Primary Standard Dosimetry Lab (PSDL) and Accredited Dosimetry Calibration Labs (ADCL) •Excellent reference class instruments. Reproducibility of the order of 0.5% or better •High precision and accuracy •Large selection of active volumes, vendors and models commercially available •Charge to dose corrections well understood •Dose rate independent •Energy dependence between 50 keV and 2 MeV is relatively constant •Instant readout •Waterproof models available	•High voltage and cables required (up to 1000 V) •Relatively large volume of some models •Fragile - must be handled with care	<0.001 Gy to >1000 Gy	1 % to 5 %[5]	≈ 1 mm to 5 mm depending on physical air volume
**Radiographic Film**	•Imaging •Qualitative and quantitative dosimetry •Measurements in solid water and anthropomorphic phantoms	•Superb 2D spatial resolution •Measurement of planar dose distributions •Dose rate independent •Variety of film types with broad range of linear response to irradiation •Good measure of radiation field size and flatness and Symmetry	•Darkroom for processing required •Processing complex to control •For identical irradiation conditions response varies between film types and batches •Dose calibration against ion chamber required •Energy dependence •Sensitive to visible light •Not reusable •Great care in processing and batch calibration if used for dose calibration	0.1 – 5 Gy	2 % to 5 %	Capable of submillimeter resolution depending on the properties of the reading device
**Radiochromic Film**	•Imaging •Qualitative and quantitative dosimetry •Measurements in solid and liquid water and anthropomorphic phantoms	•Self processing •Insensitive to visible light •Tissue equivalent •Energy independent •Dose rate independent •Superb 2D spatial resolution •Measurement of planar dose distributions •Good measure of radiation field size and flatness and symmetry •Relatively easy to read with current flatbed scanners	•For identical irradiation conditions response varies between film types and batches •Dose calibration against ion chamber required •Not reusable •Great care handling film and scanner if used for dose calibration •When flatbed scanners are used, well-defined protocols must be followed to disable the scanner imaging optimization features.	0.1–200 Gy^‡^	1 % to5 %	Capable of sub-millimeter resolution depending on the properties of the reading device
**Thermo-luminescent Detectors (TLD)**	•In vivo dosimetry •Measurements in anthropomorphic and slab phantoms •Intercomparisons between centers	•Small size – point dose measurements •Multiple measurement points in a single irradiation •Various forms and compositions available •Reusable after thermal annealing	•Time consuming calibration •Delayed readout •Elaborate care for accurate readout •Signal erased during readout •For identical irradiation conditions response varies within the same batch •Light sensitivity •Fading – signal loss over time for some materials	0.0005 to 200 Gy Supralinear range > 5 Gy	1.5 % to 5 % (w/93 % confidence)[1]	Typically limited to 2 mm to 5 mm resolution depending on the physical size of the detector
**Optically Stimulated Luminescent Detectors (OSLD)**	•In vivo dosimetry •Measurements in anthropomorphic and slab phantoms •Intercomparisons between centers	•Moderate size – point dose measurements •Multiple measurement points in a single irradiation •Fast readout •Multiple readouts possible •Dose rate independent	•Sensitivity to light – light-tight requirement prior to readout •Supralinear response at high doses •Limited selection of vendors •Not recommended for dose calibration •Energy dependence	0.005–10 Gy	1.1 % to 3.7 %[2]	Typically limited to 2 mm to 5 mm resolution depending on the physical size of the detector
**Silicon Diodes**	•In vivo dosimetry •Small field dosimetry •Detector arrays •Relative dosimetry (depth dose, profiles, output)	•Moderate size – point dose measurements •Instant readout •Great sensitivity relative to ion chambers •No external bias voltage	•Connecting cables required •Variability of calibration with temperature •Directional dependence •Special care needed for constancy of response •Cannot be used for dose calibration •Changes in sensitivity with high dose accumulation	0.005 – 10 Gy	3 % to 5 %	Capable of ≈0.5 mm resolution while maintaining adequate sensitivity
**Metal Oxide Semiconductors Field Effect Transistors (MOSFET)**	•In vivo dosimetry •Small field dosimetry •Detector arrays	•Small size – point dose measurements •Multiple measurement points in a single irradiation •Great sensitivity compared to ion chambers •Fast readout	•Calibration needed for every dosimeter •Energy dependence •Temperature dependence •Directional dependence •Not to be used for dose calibration	0.005 – 10 Gy	3 % to 5 %	Capable of ≈0.5 mm resolution while maintaining adequate sensitivity
**Diamond Detectors**	•In vivo dosimetry •Small field dosimetry •Relative dosimetry (depth dose, profiles, output)	•Small size – point dose measurements •Tissue equivalent •High sensitivity •Resistance to radiation damage	•Bias voltage and cables required •Require pre-irradiation •Variability among dosimeters •Not recommended for dose calibration •Hard to obtain	0.005 – 10 Gy	1.3 % to 3 %	≈5 mm
**Alanine – Electron Paramagnetic Resonance Detectors**	•In vivo dosimetry •Measurements in anthropomorphic and slab phantoms •Intercomparisons between centers	•Tissue equivalent •Readout non-destructive •No fading	•Dose readout requires special equipment or must be done by a primary laboratory	10–150,000 Gy	1.5 % to 4 %[4]	≈0.2 mm to 5 mm
**Gel Dosimetry Detectors**	•Measurements in complex geometries •Intercomparisons between centers	•Tissue equivalent •Gel acts as both phantom and dosimeter •True 3D dose distribution	•Complex preparation and evaluation •Post-irradiation diffusion of ions and polymerization •Limited accuracy and reproducibility •Not to be used for dose calibration	0.005 – 10 Gy	5 % to 10 %	Typically tens of cm

## 12. Appendix

- I) Examples of dosimetry content that are considered as either mandatory or strongly recommended for radiobiology publications.
  When page limitations preclude the hard copy publication of all of this information, details could be included in supplementary sections that are archived by the journal or by reference to published SOPs.
  - Absolute Dosimetry/Calibration of the beam **(Mandatory)**
    ○ published standards/protocols used for calibration geometry/set-up for calibration (if not clearly mandated by the protocol) including energy, field size, calibration point, source to detector distance
    ○ detector type used for system calibration
  - Determination of the absorbed dose within subject **(Mandatory)**
    ○ published standards/protocols used for relative dosimetry (to determine relationship between dose for the calibration field and the field size of interest at the depth of interest), if applicable
    ○ specification if doses are in water, plastic, air or *in vivo*, and if they account for animal-specific heterogeneities, etc.
    ○ detector type used
  - Radiation Source Specification **(Mandatory)**
    ○ if radioactive source (Cs-137, Co-60, Ir-192, other radionuclide)
    ○ if x-ray source, then beam quality, which includes kV setting, filter materials and thicknesses, measured half-value layer (HVL)
  - Details of the Irradiation **(Mandatory)**
    ○ animal/cell type
    ○ absorbed dose delivered (as opposed to outdated “exposure” in air), dose-rate, dose-fractionation scheme, dose-specification (dose-to-water *vs* dose-to-tissue), dose-specification point (depth)
    ○ field size and shape at subject o geometry of radiation fields (single beam, multiple beams, arc delivery, co-planar, non-coplanar etc.); photographs and/or radiographs are helpful
    ○ animal containment (if proximal, distal, or immediately lateral to the radiation path), i.e., bolus, backscatter
  - Further Details of the Irradiation **(Strongly Recommended)**
    ○ dose uniformity throughout the volume of interest and method of measuring this ○ method of field shaping (surface collimators, collimators placed at distance of *x* cm away from the subject, etc.)
    ○ factors which contribute to uncertainty in the dose (backscatter from lead or containment devices), differences between calibration conditions and irradiation geometry used (i.e., amount of backscatter or lateral scatter, tissue heterogeneity etc.)
    ○ estimated uncertainty in the absorbed dose values presented
- Example of Experimental Description for Purpose of Publication

The following is an example of the description of a hypothetical radiobiological experiment, which follows the guidelines set out in Appendix I.

The XYZ system was used for irradiation of 6-week old NOD SCID mice. The animals were irradiated with 100 kVp x-rays, HVL of 3.0 mm Al, 2 mm Al added filtration. The beam was calibrated following the AAPM TG-61 protocol, with measurement done in air, for a 10 cm × 10 cm field size at a source-to-axis distance (SAD) of 30 cm. A NE2571 ion chamber was used which was calibrated at the National Institute of Standards and Technology (NIST) in terms of air kerma (mGy/nC).

Partial-brain irradiation was delivered using opposed lateral-beam geometry, collimated to produce a 0.5 cm diameter field at the depth of interest. The source-to-axis distance was 30 cm, and the collimator-surface distance was 7 cm. A dose of 10.0 Gy in 2 fractions was delivered at a dose-rate of 2.7 Gy/min. The dose was specified at a depth of 0.5 cm, dose-to-water. The relative dosimetry was measured in water-equivalent plastic, using EBT-2 Gafchromic film. The uncertainty in the 10.0 Gy absorbed dose value was calculated to be ± 5.5 %. The animal was immobilized with a bite-bar, and anesthetized using inhaled isoflurane (delivered through a nose cone). None of the immobilization devices were in the beam path. The doses were not corrected for surface curvature or tissue heterogeneities. The dose uniformity within the radiation field was ± 5 %, and the fall-off of radiation with depth was approximately 3 % per mm of water-equivalent material. Both of these were measured using EBT-2 Gafchromic film in a water-equivalent plastic phantom.
